# [Retracted] MicroRNA-103 modulates tumor progression by targeting KLF7 in non-small cell lung cancer

**DOI:** 10.3892/ijmm.2025.5695

**Published:** 2025-11-17

**Authors:** Ke Li, Conghu Yuan

Int J Mol Med 46: 1013-1028, 2020; DOI: 10.3892/ijmm.2020.4649

Following the publication of this paper, it was drawn to the Editor's attention by a concerned reader that most of the flow cytometric data shown in Fig. 5A on p. 1020 were strikingly similar to data which had been already been submitted for publication to several other journals that were written by different authors at different research institutes.

Owing to the fact that the contentious data in the above article were found to be strikingly similar to data that had already been submitted, or accepted, for publication elsewhere, the Editor of *International Journal of Molecular Medicine* has decided that this paper should be retracted from the Journal. The authors were asked for an explanation to account for these concerns, but the Editorial Office did not receive a reply. The Editor apologizes to the readership for any inconvenience caused.

